# Supplementary material for: High enzyme promiscuity in lignin degradation mechanisms in Rhodopseudomonas palustris CGA009
Source: Appl Environ Microbiol. 2025 Jul 8;91(8):e00573-25. doi: 10.1128/aem.00573-25 (PMC12366328; doi:10.1128/aem.00573-25)
Supplement: Supplemental material — Tables S1 to S6; Fig. S1 to S5. [file aem.00573-25-s0007.docx]

**SUPPLEMENTAL MATERIAL**

**High Enzyme Promiscuity in Lignin Degradation Mechanisms in**

**Rhodopseudomonas palustris CGA009**

Mark Kathol, Niaz Chowdhury, Cheryl Immethun, Adil Alsiyabi, Dianna Morris, Michael J. Naldrett, Rajib Saha

**Supplementary Table 1.** p-values for anaerobic growth profiles max OD_660_ (vs Acetate)

| **Carbon Source** | **p-Value** |
| --- | --- |
| p-Coumarate | 0.0045 |
| Coniferyl Alcohol + Acetate | 0.0000 |
| Kraft Lignin + Acetate | 0.0019 |
| Sinapyl Alcohol + Acetate | 0.0066 |
| Sodium Ferulate + Acetate | 0.0041 |
| p-Coumaryl Alcohol + Acetate | 0.0010 |

Green highlights represent at least one level of significant difference from the corresponding aerobic acetate maximum OD_660_. Growth profiles for the anaerobic condition, as opposed to the aerobic condition, have differing lag phases and growth times, making comparison between maximum final OD_660_ a more fair comparison than at individual time points.

**Supplementary Table 2.** p-values for Aerobic Growth Profiles (vs Acetate)

| **Elapsed Time (h)** | **p-Coumarate** | **p-Coumaryl Alcohol + Acetate** | **Sodium Ferulate + Acetate** | **Coniferyl Alcohol** | **Sinapyl Alcohol** | **Kraft Lignin** |
| --- | --- | --- | --- | --- | --- | --- |
| 0 | 0.1050 | 0.5535 | 0.0132 | 0.1241 | 0.5535 | 0.0055 |
| 4 | 0.1399 | 0.0129 | 0.0497 | 0.2201 | 0.0202 | 0.0361 |
| 8 | 0.0112 | 0.0033 | 0.1893 | 0.2320 | 0.0094 | 0.1407 |
| 12 | 0.0132 | 0.0100 | 0.1797 | 0.1955 | 0.0229 | 0.0503 |
| 16 | 0.0094 | 0.0052 | 0.0465 | 0.7366 | 0.0092 | 0.0067 |
| 20 | 0.0022 | 0.0006 | 0.0416 | 0.9910 | 0.0264 | 0.0356 |
| 24 | 0.0142 | 0.0251 | 0.0471 | 0.9855 | 0.1539 | 0.0967 |
| 28 | 0.0084 | 0.0290 | 0.0373 | 0.0769 | 0.1307 | 0.2843 |
| 32 | 0.0052 | 0.0160 | 0.0276 | 0.0244 | 0.0849 | N/A |
| 36 | 0.0105 | 0.0401 | 0.0258 | 0.0109 | 0.0517 | N/A |
| 40 | 0.0135 | 0.0267 | 0.0249 | 0.0064 | 0.0293 | N/A |
| 44 | 0.0139 | 0.0169 | 0.0060 | 0.0012 | 0.0253 | N/A |
| 48 | 0.0127 | 0.0220 | 0.0082 | 0.0005 | 0.0257 | N/A |
| 52 | 0.0137 | 0.0347 | 0.0083 | 0.0005 | 0.0350 | N/A |
| 56 | 0.0127 | 0.0329 | 0.0079 | 0.0004 | 0.0208 | N/A |
| 60 | 0.0130 | 0.0334 | 0.0082 | 0.0004 | 0.0312 | N/A |

Green highlights represent at least one level of significant difference from corresponding aerobic acetate growth profiles. The sinapyl alcohol curve is temporarily statistically indistinguishable from the base acetate growth curve until the diauxic growth phase begins (40 hours).

| **Name** | **Parts** | **AddGene ID** | **Type and Source** |
| --- | --- | --- | --- |
| BBR1-kan-LacI | pBBR1 replicon; *kan^R^; P_Lac_:lacI,* | N/A | Replicative  This Study |
| BBR1-kan-dCas9-ControlVector | pBBR1 replicon; *kan^R^; P_Lac_:lacI, dCas9, rrnB, T7Te, J23119, sgRNA scaffold, tonB* | N/A | Replicative  This Study |
| BBR1-kan-dCas9- RPA0650 | pBBR1 replicon; *kan^R^; P_Lac_:lacI, dCas9, rrnB, T7Te, J23119, RPA0650sgRNA*, *sgRNA scaffold, tonB* | 240487 | Replicative  This Study |
| BBR1-kan-dCas9- RPA1206 | pBBR1 replicon; *kan^R^; P_Lac_:lacI, dCas9, rrnB, T7Te, J23119, RPA1206sgRNA, sgRNA scaffold, tonB* |  | Replicative  This Study |
| BBR1-kan-dCas9- RPA1580 | pBBR1 replicon; *kan^R^; P_Lac_:lacI, dCas9, rrnB, T7Te, J23119, RPA1580sgRNA, sgRNA scaffold, tonB* | 240488 | Replicative  This Study |
| BBR1-kan-dCas9- RPA1786 | pBBR1 replicon; *kan^R^; P_Lac_:lacI, dCas9, rrnB, T7Te, J23119, RPA1786sgRNA, sgRNA scaffold, tonB* | 240489 | Replicative  This Study |
| BBR1-kan-dCas9- RPA3701 | pBBR1 replicon; *kan^R^; P_Lac_:lacI, dCas9, rrnB, T7Te, J23119, RPA3701sgRNA, sgRNA scaffold, tonB* | 240490 | Replicative  This Study |
| BBR1-kan-dCas9- RPA3717 | pBBR1 replicon; *kan^R^; P_Lac_:lacI, dCas9, rrnB, T7Te, J23119, RPA3717sgRNA, sgRNA scaffold, tonB* | 240491 | Replicative  This Study |
| BBR1-kan-dCas9- RPA4308 | pBBR1 replicon; *kan^R^; P_Lac_:lacI, dCas9, rrnB, T7Te, J23119, RPA4308sgRNA, sgRNA scaffold, tonB* | 240492 | Replicative  This Study |
| BBR1-kan-dCas9- RPA4696 | pBBR1 replicon; *kan^R^; P_Lac_:lacI, dCas9, rrnB, T7Te, J23119, RPA4696sgRNA, sgRNA scaffold, tonB* | 240493 | Replicative  This Study |

**Supplementary Table 3.** Plasmids used in this work.

**Supplementary Table 4.**  List of genetic parts used in this work^a^.

| Part name | Type and source | DNA sequence |
| --- | --- | --- |
| *kan^R^* | Antibiotic resistance gene  (Kovach et al., 1995) | atgattgaacaagatggattgcacgcaggttctccggccgcttgggtggagaggctattcggctatgactgggcacaacagacaatcggctgctctgatgccgccgtgttccggctgtcagcgcaggggcgcccggttctttttgtcaagaccgacctgtccggtgccctgaatgaactgcaggacgaggcagcgcggctatcgtggctggccacgacgggcgttccttgcgcagctgtgctcgacgttgtcactgaagcgggaagggactggctgctattgggcgaagtgccggggcaggatctcctgtcatctcaccttgctcctgccgagaaagtatccatcatggctgatgcaatgcggcggctgcatacgcttgatccggctacctgcccattcgaccaccaagcgaaacatcgcatcgagcgagcacgtactcggatggaagccggtcttgtcgatcaggatgatctggacgaagagcatcaggggctcgcgccagccgaactgttcgccaggctcaaggcgcgcatgcccgacggcgaggatctcgtcgtgacccatggcgatgcctgcttgccgaatatcatggtggaaaatggccgcttttctggattcatcgactgtggccggctgggtgtggcggaccgctatcaggacatagcgttggctacccgtgatattgctgaagagcttggcggcgaatgggctgaccgcttcctcgtgctttacggtatcgccgctcccgattcgcagcgcatcgccttctatcgccttcttgacgagttcttctga |
| *P_Lac_* | Promoter  (Kovach et al., 1995) | ggcagtgagcgcaacgcaattaatgtgagttagctcactcattaggcaccccaggctttacactttatgcttccggctcgtatgttgtgtggaattgtgagcggataacaat |
| *dCas9* | AddGene | atggataagaaatactcaataggcttagctatcggcacaaatagcgtcggatgggcggtgatcactgatgaatataaggttccgtctaaaaagttcaaggttctggaaaatacagaccgccacagtatcaaaaaaaatcttataggggctcttttatttgacagtggagagacagcggaagcgactcgtctcaaacggacagctcgtagaaggtatacacgtcggaagaatcgtatttgttatctacaggagattttttcaaatgagatggcgaaagtagatgatagtttctttcatcgacttgaagagtcttttttggtggaagaagacaagaagcatgaacgtcatcctatttttggaaatatagtagatgaagttgcttatcatgagaaatatccaactatctatcatctgcgaaaaaaattggtagattctactgataaagcggatttgcgcttaatctatttggccttagcgcatatgattaagtttcgtggtcattttttgattgagggagatttaaatcctgataatagtgatgtggacaaactatttatccagttggtacaaacctacaatcaattatttgaagaaaaccctattaacgcaagtggagtagatgctaaagcgattctttctgcacgattgagtaaatcaagacgattagaaaatctcattgctcagctccccggtgagaagaaaaatggcttatttgggaatctcattgctttgtcattgggtttgacccctaattttaaatcaaattttgatttggcagaagatgctaaattacagctttcaaaagatacttacgatgatgatttagataatttattggcgcaaattggagatcaatatgctgatttgtttttggcagctaagaatttatcagatgctattttactttcagatatcctaagagtaaatactgaaataactaaggctcccctatcagcttcaatgattaaacgctacgatgaacatcatcaagacttgactcttttaaaagctttagttcgacaacaacttccagaaaagtataaagaaatcttttttgatcaatcaaaaaacggatatgcaggttatattgatgggggagctagccaagaagaattttataaatttatcaaaccaattttagaaaaaatggatggtactgaggaattattggtgaaactaaatcgtgaagatttgctgcgcaagcaacggacctttgacaacggctctattccccatcaaattcacttgggtgagctgcatgctattttgagaagacaagaagacttttatccatttttaaaagacaatcgtgagaagattgaaaaaatcttgacttttcgaattccttattatgttggtccattggcgcgtggcaatagtcgttttgcatggatgactcggaagtctgaagaaacaattaccccatggaattttgaagaagttgtcgataaaggtgcttcagctcaatcatttattgaacgcatgacaaactttgataaaaatcttccaaatgaaaaagtactaccaaaacatagtttgctttatgagtattttacggtttataacgaattgacaaaggtcaaatatgttactgaaggaatgcgaaaaccagcatttctttcaggtgaacagaagaaagccattgttgatttactcttcaaaacaaatcgaaaagtaaccgttaagcaattaaaagaagattatttcaaaaaaatagaatgttttgatagtgttgaaatttcaggagttgaagatagatttaatgcttcattaggtacctaccatgatttgctaaaaattattaaagataaagattttttggataatgaagaaaatgaagatatcttagaggatattgttttaacattgaccttatttgaagatagggagatgattgaggaaagacttaaaacatatgctcacctctttgatgataaggtgatgaaacagcttaaacgtcgccgttatactggttggggacgtttgtctcgaaaattgattaatggtattagggataagcaatctggcaaaacaatattagattttttgaaatcagatggttttgccaatcgcaattttatgcagctgatccatgatgatagtttgacatttaaagaagacattcaaaaagcacaagtgtctggacaaggcgatagtttacatgaacatattgcaaatttagctggtagccctgctattaaaaaaggtattttacagactgtaaaagttgttgatgaattggtcaaagtaatggggcggcataagccagaaaatatcgttattgaaatggcacgtgaaaatcagacaactcaaaagggccagaaaaattcgcgagagcgtatgaaacgaatcgaagaaggtatcaaagaattaggaagtcagattcttaaagagcatcctgttgaaaatactcaattgcaaaatgaaaagctctatctctattatctccaaaatggaagagacatgtatgtggaccaagaattagatattaatcgtttaagtgattatgatgtcgatgccattgttccacaaagtttccttaaagacgattcaatagacaataaggtcttaacgcgttctgataaaaatcgtggtaaatcggataacgttccaagtgaagaagtagtcaaaaagatgaaaaactattggagacaacttctaaacgccaagttaatcactcaacgtaagtttgataatttaacgaaagctgaacgtggaggtttgagtgaacttgataaagctggttttatcaaacgccaattggttgaaactcgccaaatcactaagcatgtggcacaaattttggatagtcgcatgaatactaaatacgatgaaaatgataaacttattcgagaggttaaagtgattaccttaaaatctaaattagtttctgacttccgaaaagatttccaattctataaagtacgtgagattaacaattaccatcatgcccatgatgcgtatctaaatgccgtcgttggaactgctttgattaagaaatatccaaaacttgaatcggagtttgtctatggtgattataaagtttatgatgttcgtaaaatgattgctaagtctgagcaagaaataggcaaagcaaccgcaaaatatttcttttactctaatatcatgaacttcttcaaaacagaaattacacttgcaaatggagagattcgcaaacgccctctaatcgaaactaatggggaaactggagaaattgtctgggataaagggcgagattttgccacagtgcgcaaagtattgtccatgccccaagtcaatattgtcaagaaaacagaagtacagacaggcggattctccaaggagtcaattttaccaaaaagaaattcggacaagcttattgctcgtaaaaaagactgggatccaaaaaaatatggtggttttgatagtccaacggtagcttattcagtcctagtggttgctaaggtggaaaaagggaaatcgaagaagttaaaatccgttaaagagttactagggatcacaattatggaaagaagttcctttgaaaaaaatccgattgactttttagaagctaaaggatataaggaagttaaaaaagacttaatcattaaactacctaaatatagtctttttgagttagaaaacggtcgtaaacggatgctggctagtgccggagaattacaaaaaggaaatgagctggctctgccaagcaaatatgtgaattttttatatttagctagtcattatgaaaagttgaagggtagtccagaagataacgaacaaaaacaattgtttgtggagcagcataagcattatttagatgagattattgagcaaatcagtgaattttctaagcgtgttattttagcagatgccaatttagataaagttcttagtgcatataacaaacatagagacaaaccaatacgtgaacaagcagaaaatattattcatttatttacgttgacgaatcttggagctcccgctgcttttaaatattttgatacaacaattgatcgtaaacgatatacgtctacaaaagaagttttagatgccactcttatccatcaatccatcactggtctttatgaaacacgcattgatttgagtcagctaggaggtgactga |
| *pLacI* | AddGene | attcaccaccctgaattgactctcttccgggcgctatcatgccataccgcgaaaggttttgcgccattcgatggtgtc |
| *LacI* | AddGene | tcactgcccgctttccagtcgggaaacctgtcgtgccagctgcattaatgaatcggccaatgcgcggggagaggcggtttgcgtattgggcgccagggtggtttttcttttcaccagtgagacgggcaacagctgattgcccttcaccgcctggccctgagagagttgcagcaagcggtccacgctggtttgccccagcaggcgaaaatcctgtttgatggtggttaacggcgggatataacatgagctgtcttcggtatcgtcgtatcccactaccgagatgtccgcaccaacgcgcagcccggactcggtaatggcgcgcattgcgcccagcgccatctgatcgttggcaaccagcatcgcagtgggaacgatgccctcattcagcatttgcatggtttgttgaaaaccggacatggcactccagtcgccttcccgttccgctatcggctgaatttgattgcgagtgagatatttatgccagccagccagacgcagacgcgccgagacagaacttaatgggcccgctaacagcgcgatttgctggtgacccaatgcgaccagatgctccacgcccagtcgcgtaccgtcttcatgggagaaaataatactgttgatgggtgtctggtcagagacatcaagaaataacgccggaacattagtgcaggcagcttccacagcaatggcatcctggtcatccagcggatagttaatgatcagcccactgacgcgttgcgcgagaagattgtgcaccgccgctttacaggcttcgacgccgcttcgttctaccatcgacaccaccacgctggcacccagttgatcggcgcgagatttaatcgccgcgacaatttgcgacggcgcgtgcagggccagactggaggtggcaacgccaatcagcaacgactgtttgcccgccagttgttgtgccacgcggttgggaatgtaattcagctccgccatcgccgcttccactttttcccgcgttttcgcagaaacgtggctggcctggttcaccacgcgggaaacggtctgataagagacaccggcatactctgcgacatcgtataacgttactggtttcac |
| *pLac* | AddGene | tttacactttatgcttccggctcgtatgttg |
| *Lac operator* | AddGene | ttgtgagcggataacaa |
| *rrnB* | AddGene | caaataaaacgaaaggctcagtcgaaagactgggcctttcgttttatctgttgtttgtcggtgaacgctctc |
| *T7Te* | AddGene | ggctcaccttcgggtgggcctttctgcg |
| *J23119* | AddGene | ttgacagctagctcagtcctaggtataatgctagc |
| *sgRNA scaffold* | AddGene | gttttagagctagaaatagcaagttaaaataaggctagtccgttatcaacttgaaaaagtggcaccgagtcggtgc |
| *tonB* | AddGene | agtcaaaagcctccgaccggaggcttttgact |

^a^ <http://www.ncbi.nlm.nih.gov/gene>

**Supplementary Table 5.**  sgRNA sequences for targeted dCas9 suppression.

| Primer Name | DNA Sequence | Predicted Suppression | | | Log_2_ Fold Repression | Purpose |
| --- | --- | --- | --- | --- | --- | --- |
| *RPA0650* | CCTATCCTCACCGAAACCCAGGG | | 67.01% | 5.78 | | Redox |
| *RPA1206* | CCAGATGCGGATCTTCCAGGAGG | | 73.93% | nd | | Catabolism |
| *RPA1580* | CCGTGCCTCAGAGCTCACCGAGG | | 78.66% | 3.815 | | Redox |
| *RPA1786* | GCGCCCTGTCAGCATCATGTCGG | | 69.57% | 2.88 | | Catabolism |
| *RPA3701* | CTTTTACACCATGAACCGCGCGG | | 72.61% | -0.26 | | Redox |
| *RPA3717* | GTGTACCCGATGATCAACGAGGG | | 75.26% | 2.14 | | Redox |
| *RPA4308* | ATCCTTGAAGATCTGCACGGCGG | | 79.52% | 1.99 | | Redox |
| *RPA4696* | TCGGTGCGAAACATCACCAGCGG | | 75.60% | 1.06 | | Catabolism |

^a^ https://chopchop.cbu.uib.no/

**Supplementary Table 6.** RT-qPCR primers.

| **Primer**  **Name** | **Sequence** | **Amplicon Length (bp)** | **Primer Concentration (nM)** | **cDNA or gDNA Concentration (ng/μL)** | **Primer**  **Efficiency**  **(%)** | **R^2** |
| --- | --- | --- | --- | --- | --- | --- |
| 16SrRNA F | GCTTAACACAT  GCAAGTCGAAC | 230 | 40 | 0.05 | 98.61 | 0.994 |
| 16SrRNA R2 | TCATCCTCTCA  GACCAGCTAC |  |  |  |  |  |
| *RPA0650 F3* | GATCGCTATGGTCTGGTCTCC | 128 | 50 | 100 | 102.26 | 0.989 |
| *RPA0650 R* | CGATTGAGGCTTCCTTCAGC |  |  |  |  |  |
| *RPA1206 F* | GCTCGACAAGATCCTGTCCTACATC | 169 | 50 | 100 | nd | NA |
| *RPA1206 R* | GAAGATCTCCTCCTGGAAGATCC |  |  |  |  |  |
| *RPA1580 F3* | CTGATCGGCAATCACTTCGTG | 101 | 50 | 100 | 99.15 | 0.977 |
| *RPA1580 R3* | AGCATGTCGTAAGAATAGCGAGAGC |  |  |  |  |  |
| *RPA1786 F2* | CTACAGGCGTTGCCGATGATC | 136 | 50 | 100 | 106.28 | 0.983 |
| *RPA1786 R* | TGGCTGTCTTGTGATCGAGGAAAG |  |  |  |  |  |
| *RPA3701 F3* | CATCACCCAGTTCTTCTTCGATAACG | 119 | 50 | 100 | 100.15 | 0.997 |
| *RPA3701 R* | CCTGCTTGAAGTTCTGCACC |  |  |  |  |  |
| *RPA3717 F* | GTCGAACACCTCGTATCTGTCGATC | 115 | 50 | 100 | 101.35 | 0.986 |
| *RPA3717 R2* | GATTTCGCACAGCTTCATCACGTTG |  |  |  |  |  |
| *RPA4308 F* | GTTCGACGTGTTCTCTGAAGAGC | 153 | 50 | 100 | 102.21 | 0.984 |
| *RPA4308 R* | GTCAGCAGATAGTCGCTCATCTG |  |  |  |  |  |
| *RPA4696 F2* | GAGGTGACCTGCATCGACAAC | 136 | 50 | 100 | 97.712 | 1 |
| *RPA4696 R* | CGATCCGCAGTTTTTCCAGC |  |  |  |  |  |

**Supplementary Figure 1.** Correlation between Cell Dry Weight and OD_660_ for aerobic cultures.

**Supplementary Figure 2.** Correlation between Cell Dry Weight and OD_660_ for anaerobic cultures. An almost perfect correlation (R^2^ > 0.99) exists between CDW and OD_660_ measurements for both aerobic and anaerobically incubated cultures, making OD_660_ a viable surrogate for measuring cell densities.


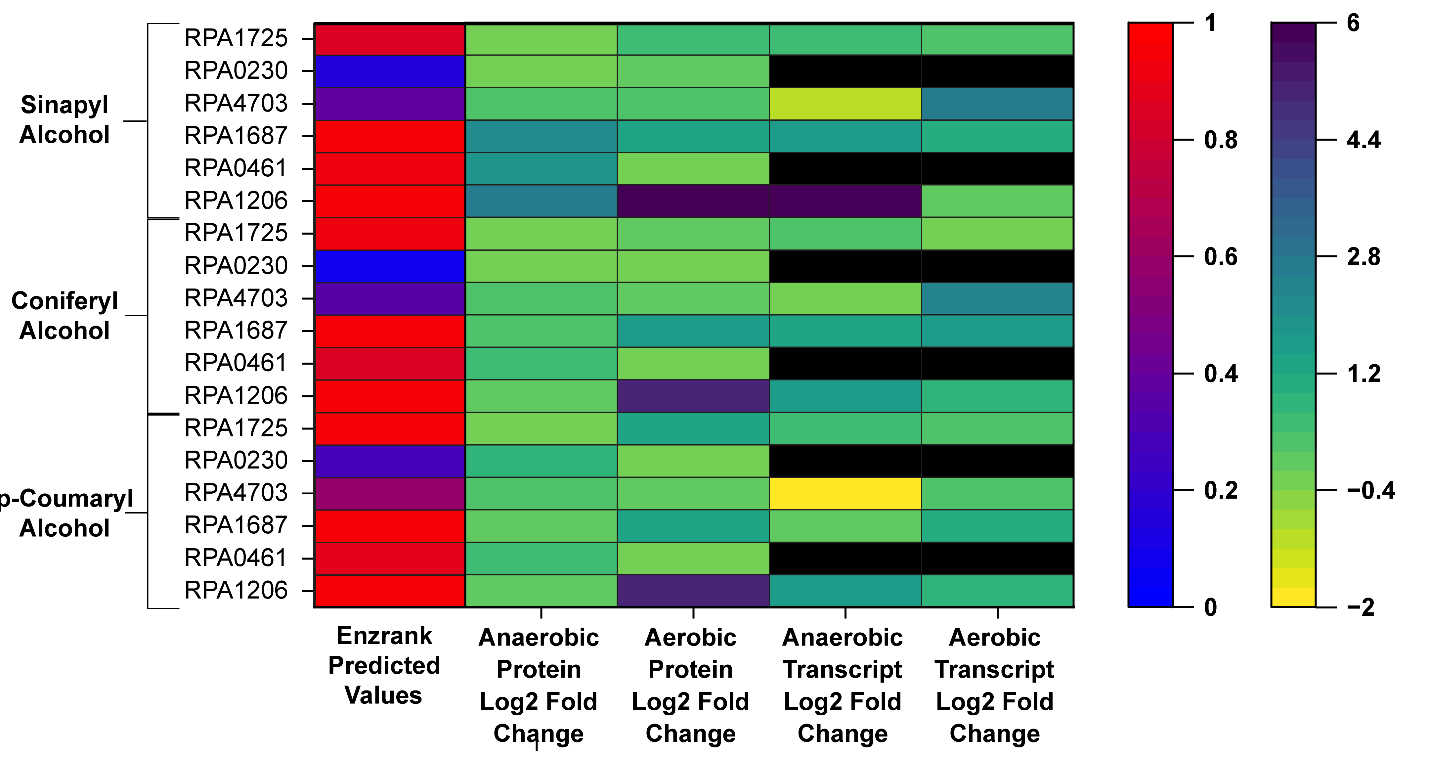


**Supplementary Figure 3.** Combined heatmap for aldehyde dehydrogenase EnzRank predictions for multiple upregulated aldehyde dehydrogenases. Predictions and upregulation states are depicted for *R. palustris* cultures grown under each type of lignin both aerobically and anaerobically.


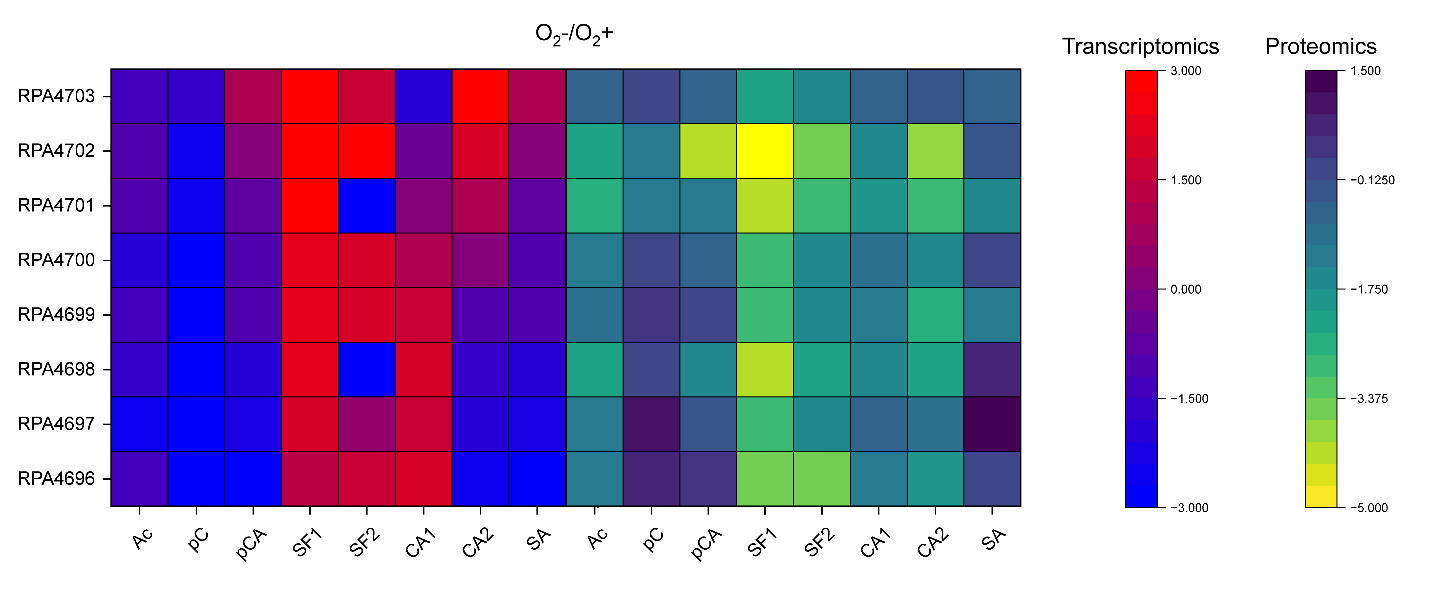


**Supplementary Figure 4.** Upregulation heatmap for meta-cleavage of anaerobic state compared to corresponding aerobic state. SF1 represents sodium ferulate only samples, while SF2 represents sodium ferulate and acetate supplemented samples. CA1 represents coniferyl alcohol only samples, while CA2 represents coniferyl alcohol and acetate supplemented samples. Meta-cleavage is seemingly upregulated under anaerobic conditions in some samples.


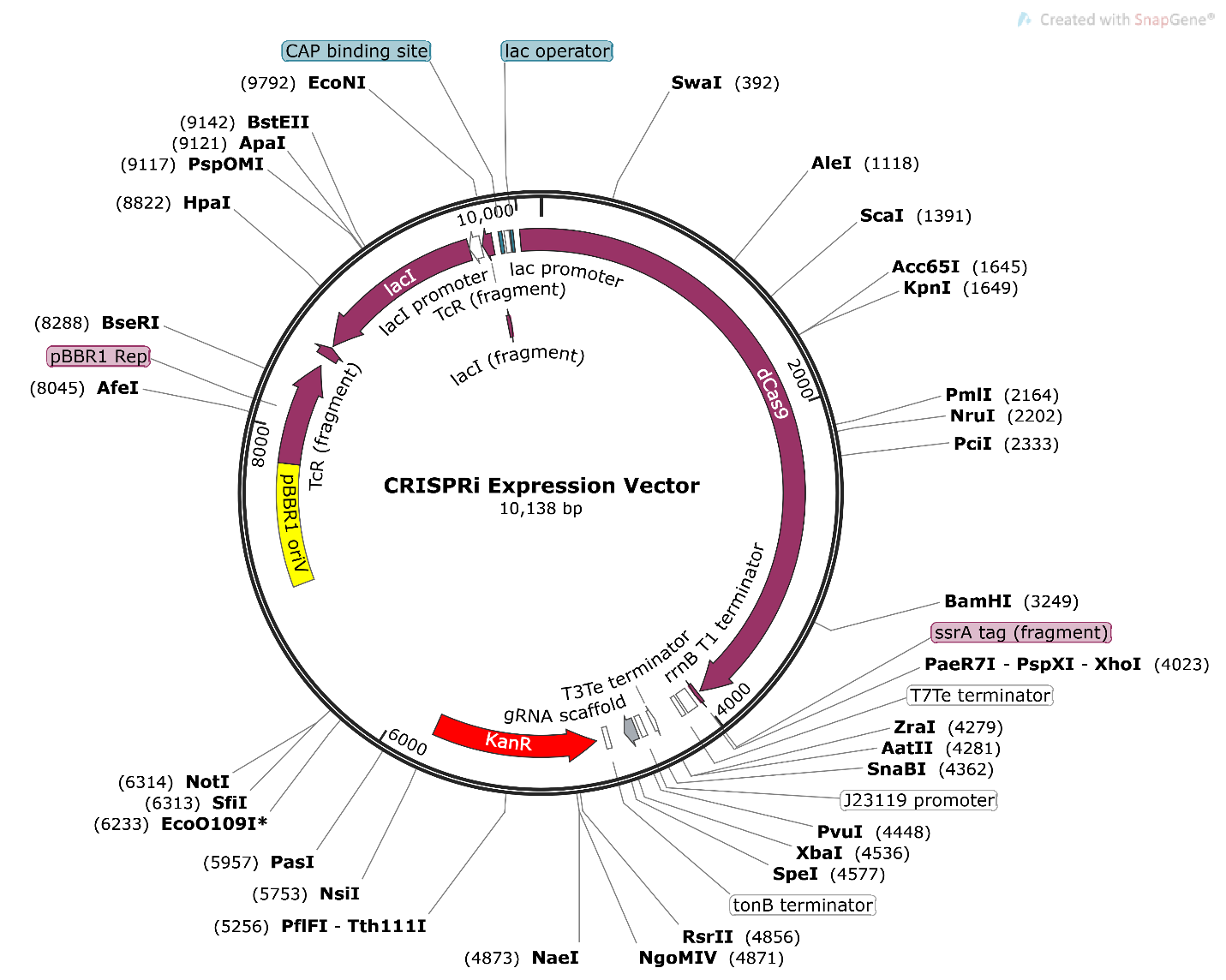


**Supplementary Figure 5.** Map of dCas9 expression vector with interchangeable sgRNA sequences.
